# Lung Collagens Perpetuate Pulmonary Fibrosis via CD204 and M2 Macrophage Activation

**DOI:** 10.1371/journal.pone.0081382

**Published:** 2013-11-20

**Authors:** Mirjam Stahl, Jonas Schupp, Benedikt Jäger, Michael Schmid, Gernot Zissel, Joachim Müller-Quernheim, Antje Prasse

**Affiliations:** Department of Pneumology, University Medical Centre, Freiburg, Freiburg, Germany; University of Pittsburgh, United States of America

## Abstract

Idiopathic pulmonary fibrosis is characterized by abundant collagen production and accumulation of alternatively activated macrophages (M2) in the lower respiratory tract. Mechanisms as to how alveolar macrophages are activated by collagen breakdown products are unknown. Alveolar macrophages were obtained by bronchoalveolar lavage from 30 patients with idiopathic pulmonary fibrosis (IPF) and 37 healthy donors (HD). Alveolar macrophages were cultured in the presence of collagen type I, III, IV and V monomers w/wo a neutralizing antibody against scavenger receptor I class A (CD204). Culture supernatants were assayed for the M2 markers CCL18, CCL2, and interleukin-1 receptor antagonist (IL-1ra) by ELISA. Furthermore, expression of phospho-Akt was measured using ELISA and expression of CD204 by RT-PCR and flow cytometry. Stimulation with collagen type I and III monomers significantly up-regulated CCL18, IL-1ra production of alveolar macrophages. Furthermore, expression of CCL2 and CD204 were up-regulated by collagen type I exposure. In addition, collagen type I stimulation increased pospho-Akt expression. Collagen type I effects were abrogated by neutralizing antiCD204 and a non-selective Phosphatidylinositide 3-kinase inhibitor (LY294002). Spontaneous CD204 expression of alveolar macrophages was significantly increased in patients with IPF. In conclusion, our findings demonstrate that monomeric collagen type I via CD204 induces phospho-Akt expression shifting alveolar macrophages to the profibrotic M2 type. Innate immune responses induced by collagen monomers might perpetuate pulmonary fibrosis.

## Introduction

Idiopathic pulmonary fibrosis (IPF) is a devastating lung disease often leading to patient death [1–3]. IPF is the most common fibrotic lung disease with a mean survival of 2 ½ years. Increased collagen type I production and accumulation is the hallmark of IPF [1,4]. Collagens build the scaffold of the human body and are the major constituent of extracellular matrix (ECM) [4]. The balance between collagen degradation and production is tightly regulated in normal tissues. Compelling evidence indicates that collagen degrading enzymes and collagen turnover are also increased in IPF, although the end result is abundant collagen type I deposition [5–7]. Native collagen type I fibrils consist of many polymerized collagen type I monomers which are covalently cross-linked. In the process of degradation, firstly the crosslinks have to be cleaved resulting in collagen type I monomers. Triple helical collagen type I monomers contain multiple cleavage sites for metalloproteinases (MMP) as well as multiple binding sites for cells, cytokines, and other extracellular matrix proteins [6,8]. However, the distinct immune response following stimulation of alveolar macrophages (AM) by collagen type I monomers and its underlying mechanisms have, to our knowledge, thus far, not been addressed. 

Macrophages have a pivotal role in wound healing and fibrosis [9–11]. Previous studies showed that CCL18 is abundantly produced by AM from patients with pulmonary fibrosis compared to AM from healthy humans [12,13]. In IPF, CCL18 production by AM correlates with CCL18 serum levels and predicts patient outcome [13,14]. CCL18 is a marker of alternative macrophage activation [15]; other markers of human alternative (M2) macrophage activation are interleukin-1 receptor antagonist (IL-1ra), CCL17, CCL22 and CD206 [15]. The phenotype of M2 macrophages has been separated in several subtypes. Recently, wound-healing macrophages have been defined as a distinct subtype of M2 macrophages [15]. Macrophages in fibrotic diseases possibly belong to this subtype [16]. It is well described that M2 cytokine production is increased following stimulation of macrophages with TH2-cytokines and IL-10 [15]. However, although TH2-cytokine and IL-10 expression is increased in various fibrotic lung diseases, in IPF neither an increase in T-cells nor in TH2 cytokines was described. Previous studies from our laboratory demonstrated that adhesion to collagen type I increases CCL18 production by AM [13]. ECM adhesion of macrophages is closely related to scavenger receptors, which are trimeric integral glycoproteins of the cell membrane whose extracellular domains consist of an alpha-helical, collagen-like, and globular structure [17]. CD204, the first identified member of class A type I scavenger receptors (SR-AI), is a homotrimeric glycoprotein of three 77kDa monomers, alternatively spliced from the product of one gene [18] and known to be expressed on macrophages. Recent evidence suggests that SR-AI plays a critical role in the induction of innate immune and inflammatory responses by recognition of exogenous PAMP and endogenous ligands [19]. There is data indicating that SR-AI is regulating LPS-induced TLR4-mediated NF-κB activation and inflammatory cytokine production in macrophages and thereby directing M1/M2 polarization [20,21].

On the background of these findings we wondered if scavenger receptors might be involved in collagen type I adhesion, and whether adhesion to collagen monomers directs macrophage activation. 

## Materials and Methods

### Study Subjects

Thirty patients with IPF, diagnosed according to the published consensus statements [22], were subjected to bronchoscopic evaluation. 37 healthy volunteers served as a control group after they gave their written informed consent. Patients were excluded when symptoms, laboratory findings or BAL cell composition suggested additional pulmonary infection. None of the 30 IPF patients received immunosuppressive treatment or antioxidants. The local ethics committee of the University Freiburg approved the study. 

### Preparation of Bronchoalveolar Lavage (BAL) Derived Cells

BAL was performed using standard technique. BAL cells were processed and cultured as previously described [13]. The isolation procedure resulted in an AM population that was 99% pure by cell morphology. AM were cultured in serum-free macrophage medium (MM, Gibco^®^, Germany), supplied with 1% penicillin/streptomycin solution, in 24-well plates (1x10^6^ cells/well and ml) for 24 hours. If indicated, cells were cultured in rat collagen type I coated plates (collagen-R, Serva, Germany, 100 µg/ml). Additional experiments were performed with human collagen type I, human collagen type III, human collagen type IV and human collagen type V. Collagen type I coating was done by preincubation of plates with collagen type I dissolved in H_2_O overnight at 37°C. In some experiments, cells were incubated with a monoclonal antibody against human SR-AI (Clone 351620) antiCD204, 2µg/ml, R&D, Germany) or mouse IgG1 prior to cell culture for 30 min, or were stimulated in the presence of LY294002 (50 μM, Invitrogen, Germany). Cell-free supernatants were harvested after 24 hours and cell pellets were lysed with TRIzol^®^ Reagent (Invitrogen, Germany). Cell-free supernatants and cell lysates were frozen and kept at -70°C before ELISA assay and RNA isolation. For FACS analysis, see cell preparation below.

### Reverse Transcription and Real Time PCR

Total RNA was extracted from 1x10^6^ cells using TRIzol^®^ Reagent according to the manufacturer‘s protocol (Invitrogen, Germany). Total RNA was reverse-transcribed with StrataScript RT (Stratagene, CA, USA) using oligo (dT)_12-18_ primer to produce cDNA according to the manufacturer‘s protocol. Specific primers for human CCL18, CD204, and GapDH were designed using Primer3 software (Whitehead Institute for Biomedical Research, Cambridge, USA; http://www.broad.mit.edu/genome_ software/other/primer3.html), Amplify1.2 software (University of Wisconsin, USA; http://engels.genetics.wisc. edu/amplify) using LocusLink and GenBank databases (National Centre for Biotechnology Information; http://www.ncbi.nlm.nih.gov/ LocusLink/index.html). The sequences of the CCL18 forward and reverse primers are 5‘-ccc tcc ttg tcc tcg tct g-3‘ and 5‘-gct tca ggt cgc tga tgt att-3‘, of CD204 right and left primers are 5’-CTC CCC TTT TCC CCT TTC TG-3’ and 5’-ATC GAG GTC CCA CTG GAG AAA GT-3’ and of GapDH forward and reverse primers are 5‘-cac cag ggc tgc ttt taa ct-3‘ and 5‘-gat ctc gcT ccT gga aga tg-3‘, respectively. All primers were intron-spanning and synthesized by MWG-Biotech (MWG-Biotech AG, Germany). Real time PCR was performed with the iQ SYBR Green SuperMix, iCycler thermocycler, and iCycler iQ 3.0 software (Bio-Rad Laboratories GmbH, Germany) according to the manufacturer‘s protocol. To control for specificity of the amplification products, a melting curve analysis was performed. No amplification of nonspecific products was observed in any of the reactions. Each sample was analysed independently in duplicate for sample and GapDH. A threshold cycle value (Ct) was calculated and used to calculate the relative level of sample mRNA by the following formula: 2^(Ct GapDH-Ct sample)^ x10,000 for each cDNA sample. 

### FACS Analysis

Freshly isolated BAL cells from healthy donors (HD) and patients with IPF were stained using goat biotinylated anti-human CD204 antibody (R&D) and streptavidin/RPE (DakoCytomation, Germany) as described [13]. Cells were analysed by flow cytometry (FACSCalibur, BD Biosciences, Germany). The percentage of positive cells was determined using isotype-matched biotinylated antibodies as a control. The relative fluorescence intensity (RFI) was calculated as the difference of the intensity of CD204 staining and the intensity of isotype matched control antibody staining.

### Cytokine and Phospho-Akt Detection by ELISA

CCL18, CCL2 and IL-1ra were quantified using a DuoSet ELISA Development System (R&D Systems Europe, UK) as described by manufacturer. 

Phospho-Akt was measured with a specific ELISA (phospho-Akt [Ser473] Pathscan Sandwich ELISA Kit; Cell Signalling Technology, Danvers, MA) in cell lysates according to the manufacturer’s protocol. 

### Analysis

Comparisons of the results between the patients’ group and controls were made using the Mann-Whitney U test. Statistical comparisons between experimental and control data for the *in vitro* studies were made by ANOVA with post hoc Fisher‘s protected least significant difference. For nonparametric analysis the Wilcoxon signed rank test was used. Either the standard correlation model or a simple regression model determined correlation. Probability values were considered significant if they were less than 0.05. 

## Results

### Collagen Monomers Increase M2 Marker and CCL2 Expression of Human AM

**Figure 1:**
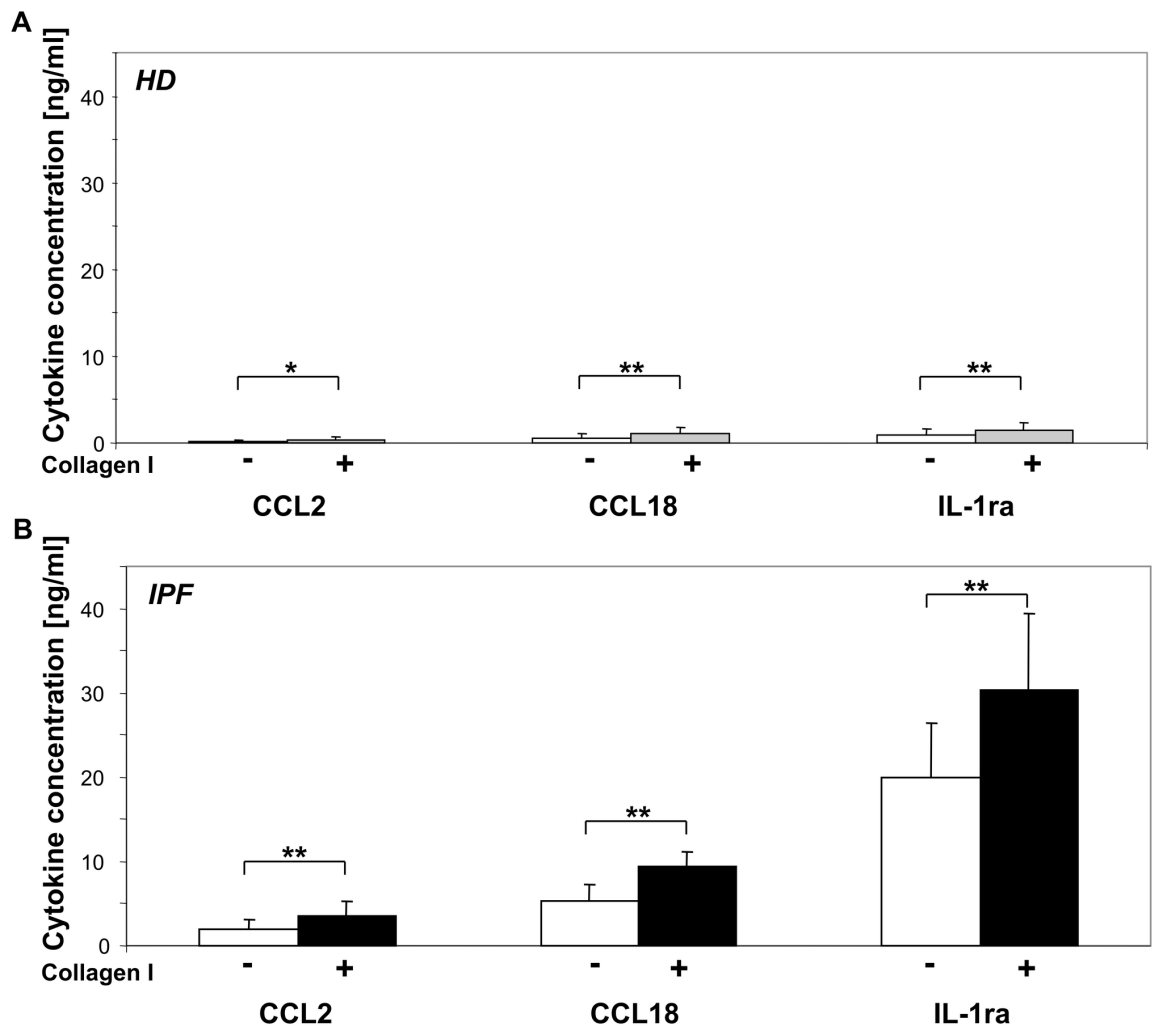
Collagen type I monomers increase M2 marker production by AM. AM (1x10^6^ cells/well and ml) from healthy donors (HD, n=23; Panel A) and IPF patients (IPF, n=14; Panel B) were cultured in serum-free medium with (coloured bar) or without (white bar) the presence of monomeric collagen type I for 24 hours. Note the 10fold difference in spontaneous M2 cytokine production between AM from IPF patients and HD. Collagen type I exposure of AM significantly enhanced CCL2, CCL18 and IL-1ra production. The effect of collagen type I monomers was more pronounced in AM from IPF. (*, *p*<0.05; **, *p*<0.001).

**Figure 2:**
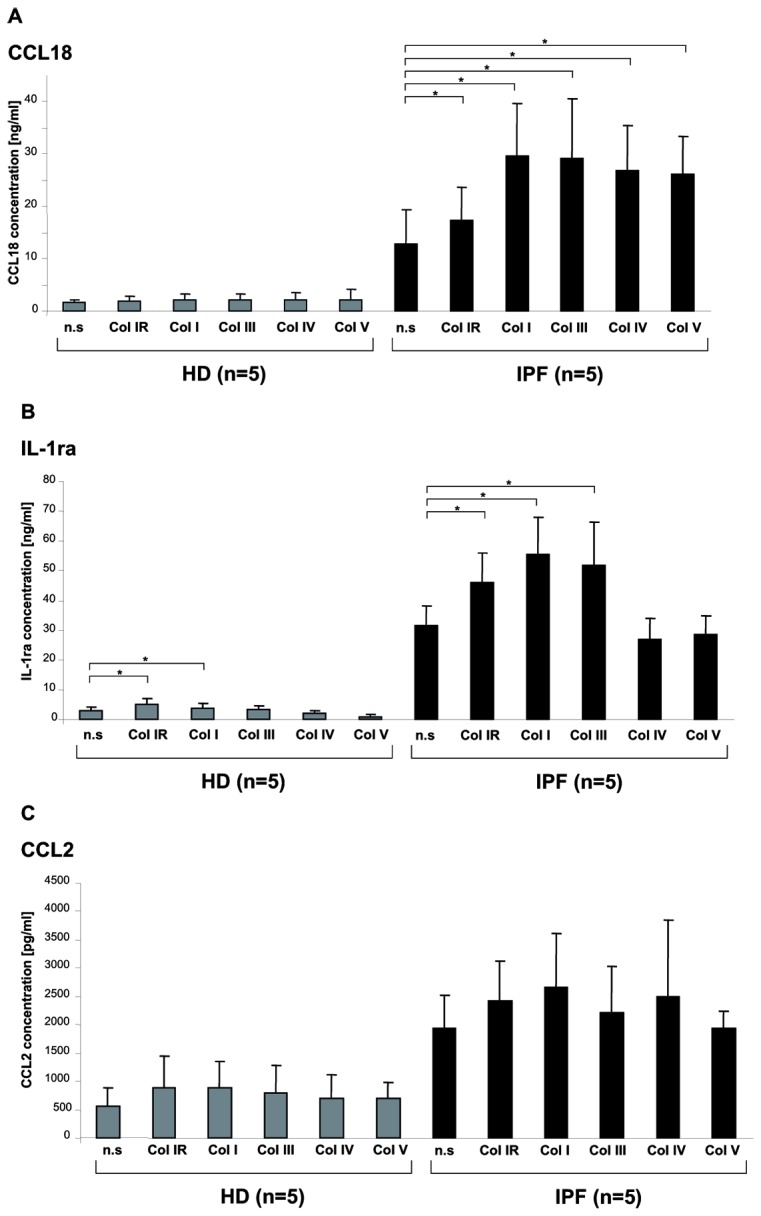
Collagen monomers increase M2 marker production by AM. M2 cytokine production of BAL cells stimulated with monomers of various collagen types. M2 cytokine production of BAL cells from 5 additional patients with IPF are depicted in black and 5 additional experiments with BAL cells from healthy donors are depicted in grey. **Panel A** shows CCL18 production following stimulation with rat collagen-I (Col 1R; 100µg/ml), human collagen-I (Col I, 100 µg/ml), human collagen-III (Col III, 100 µg/ml), human collagen-IV (Col IV, 100 µg/ml), human collagen-V (Col V, 100 µg/ml). **Panel B** shows IL-1ra production following the same stimulation protocol. **Panel C** shows CCL2 production. *p<0.05.

The direct effect of rat collagen type I monomers on M2 cytokine production by AM was tested. Monomeric rat collagen type I was produced by dissolving collagen type I in H_2_O. There was a significant increase in CCL18 protein production by collagen type I stimulated AM from healthy donors compared to non stimulated control (p<0.001; Figure 1A). Of interest, the effect of rat collagen type I stimulation on CCL18 production by AM was even more pronounced in IPF patients (p<0.001; Figure 1B). In the presence of collagen type I monomers, we also observed an increase in the production of the M2 cytokine IL-1ra and of CCL2 (Figure 1A+B), but not of CCL17 and CCL22 (data not shown). Again, the effect was more pronounced in AM from patients with IPF (CCL2: p<0.001; IL-1ra: p<0.001) compared to AM from healthy donors (CCL2: p=0.033, IL-1ra: p=0.01). In addition, the effect of collagen type I monomers on CCL18 mRNA expression by AM from patients with IPF and healthy donors was tested. AM of HD cultured serum-free for 24 hours in collagen type I coated wells expressed an about 24-fold higher amount of CCL18 mRNA than non-stimulated control (p<0.001). CCL18 mRNA expression corresponded well with protein production (data not shown). In additional experiments, similar data were also obtained for stimulation of AM with either human collagen type I monomers, human collagen type III monomers, human collagen type IV monomers and human collagen type V monomers (Figure 2).

### AM from Patients with IPF Express More CD204 mRNA and Protein Than AM from Healthy Volunteers

**Figure 3:**
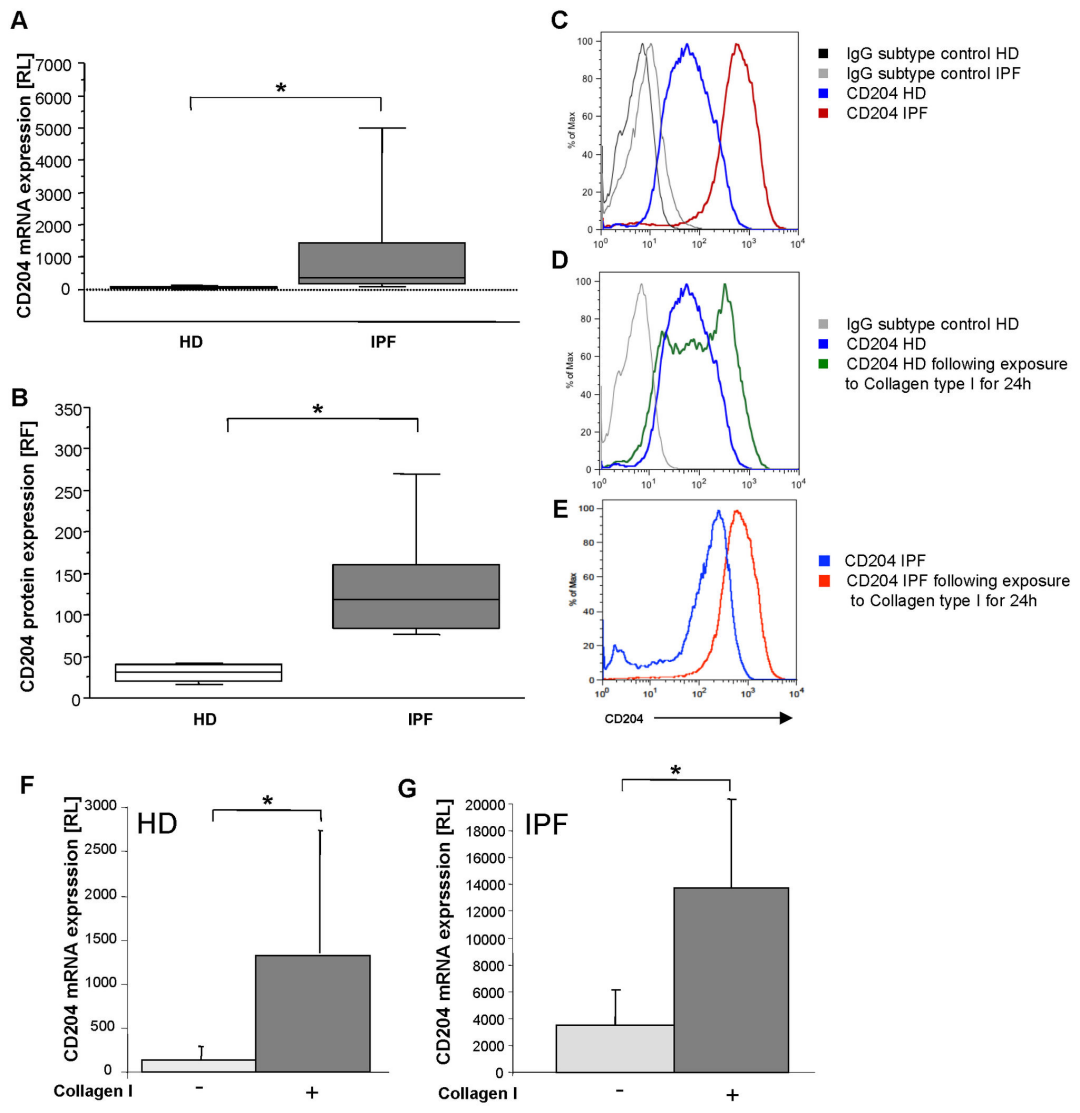
CD204 expression is up-regulated in IPF patients. In Panel **A**, relative level (RL) of CD204 mRNA expression of AM from IPF patients (n=20, grey) and healthy donors (n=18, white) is given. Similar results were obtained for CD204 relative fluorescence intensity (RFI) by flow cytometry. In Panel **B**, mean level of CD204 fluorescence intensity is depicted for naïve AM from IPF patients (n=10, grey) and healthy donors (HD, n=10, white). In Panel **C** representative original measurements of CD204 and isotype control by flow cytometry are depicted for healthy donor (HD) and patient with IPF. Exposure to collagen type I significantly increased CD204 protein and CD204 mRNA expression in AM of 5 healthy donors (Panel **D** and **F**) and IPF patients (Panel E and G). Box plots: horizontal lines represent median, 25 and 75 percentiles, and small lines characterize 10 and 90 percentiles (*, *p*<0.05; **, *p*<0.001).

The scavenger receptor I class A (CD204) is associated with collagen type I phagocytosis [17]. CD204 mRNA expression by RT-PCR of naïve AM revealed a significantly, 18-fold higher level in the relative expression of CD204 mRNA in AM from IPF patients compared to AM from healthy donors (p<0.0001; Figure 3A). Flow cytometric measurements showed that CD204 is highly expressed on AM from IPF patients and healthy donors. More than 85% of all cells expressed CD204. However, the level of CD204 expression differed between both groups: The mean RFI for CD204 on AM from IPF patients was significantly higher compared to AM from HD (p=0.033; 104 ± 78 RFI versus 40 ± 31 RFI; respectively; Figure 3B and 3C).

### AM Express More CD204 when Exposed to Collagen Type I Monomers

Stimulation with collagen type I monomers resulted in an increase in CD204 protein expression of normal alveolar macrophages (p=0.02, Figure 3D) and IPF alveolar macrophages (p=0.04, Figure 3E). In addition, we found an increase in relative CD204 mRNA expression following stimulation with collagen type I monomers compared to unstimulated controls by Real time-PCR for both normal (p<0.008; Figure 3F) and IPF macrophages (p<0.01, Figure 3G). Thus, monomeric collagen type I up-regulates one of its own receptors.

### AntiCD204 Inhibits the Stimulating Effect of Collagen Type I on the CCL18 Expression and Production by Human Alveolar Macrophages

**Figure 4:**
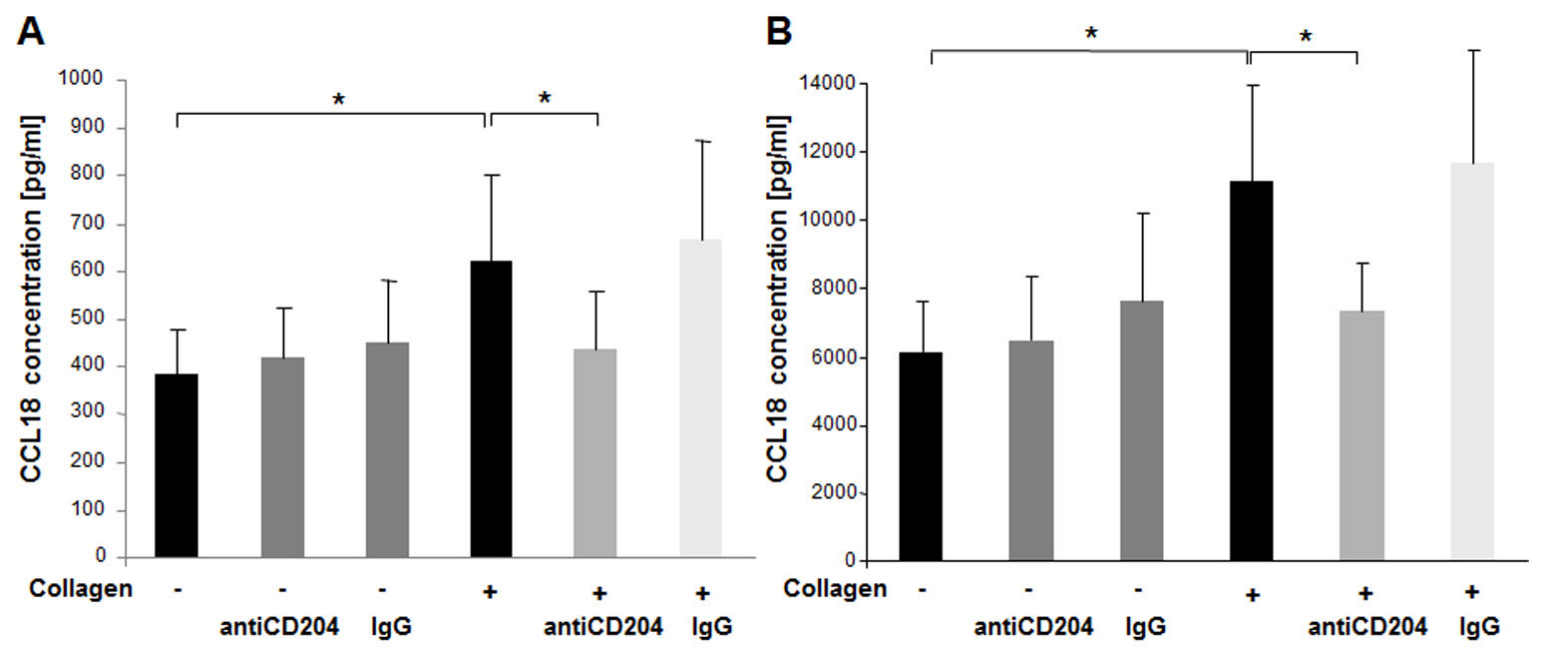
Blocking of CD204 abrogates effects mediated by collagen type I monomers. Preincubation of AM from HD (n=10, Panel A) and IPF patients (n=8, Panel B) with neutralizing antiCD204 (2µg/ml) prior to cell culture abrogated collagen type I monomers induced increase in CCL18 production. Mouse IgG1 served as control. Data are expressed by mean ± SD (*, *p*<0.05).

Scavenger receptors play important roles in macrophage binding to ECM proteins and their activation [18]. Normal AM from healthy donors were preincubated for 30 minutes with a specific blocking monoclonal antibody (mAb) for scavenger receptor I class A (antiCD204), mouse IgG1, antiCD11b, as well as antiCD36 and were then exposed to collagen type I for 24 hours. Preincubation with neutralizing antiCD204 blocked the collagen type I induced increase in CCL18 protein expression of both normal AM (p=0.02, Figure 4A) and IPF AM (p=0.01, Figure 4B), whereas preincubation with either antiCD11b or antiCD36 showed no decrease in CCL18 mRNA or protein levels (data not shown). There were no statistical significant differences in CCL18 expression between the unstimulated control, mouse IgG1 control and the cells pre-incubated with antiCD204 without collagen type I stimulation (Figure 4). Similar results were obtained measuring CCL18 mRNA expression (data not shown). Thus, the increase in CCL18 production by monomeric collagen type I stimulation of human alveolar macrophages was partially blocked by antiCD204.

### Stimulation with Collagen Type I Monomers Increased Phosphatidylinositide 3-Kinase Expression in AM

**Figure 5:**
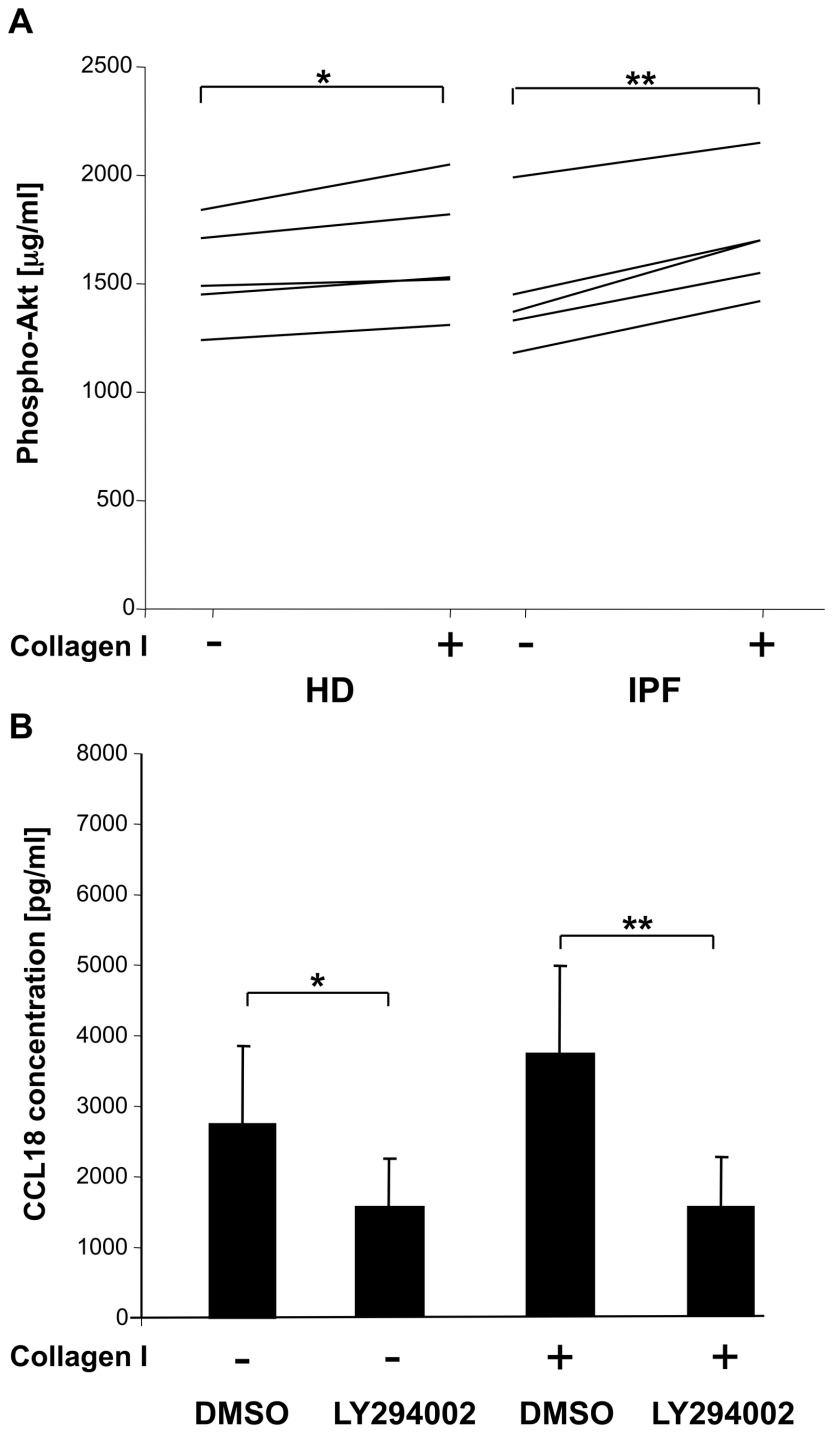
Collagen type I monomers increase Phospho-Akt expression via PI3kinase activation. Stimulation of AM with collagen type I monomers resulted in a moderate increase in phospho-Akt expression, which was more pronounced in IPF patients (Panel **A**). Basal, constitutive phospho-Akt expression was high in AM from each 5 healthy donors and IPF patients. A non-selective inhibitor of PI3kinase, LY294002 (50 μM, DMSO as control), abrogated CCL18 production following stimulation with collagen type I monomers and decreased significantly spontaneous CCL18 production of AM (Panel **B**). Data are expressed by mean ± SD (*, *p*<0.05; **, p<0.005).

M2 marker production is linked with Phosphatidylinositide 3-kinase (PI3K) activation and phospho-Akt expression. There was a high constitutive expression of phospho-Akt in AM measured by a commercially available ELISA. Stimulation of AM with collagen type I monomers resulted in a moderate increase in Pospho-Akt expression in AM from HD and AM from IPF patients (Figure 5A). 

### Inhibitor of PI3K Decreased M2 Marker Expression

The non-selective inhibitor of PI3K, LY294002 (50 μM), significantly decreased spontaneous CCL18 (p=0.02; Figure 5B), IL-1ra and CCL2 (data not shown) production by AM of healthy donors. Furthermore, LY294002 completely inhibited collagen type I monomers induced increase in CCL18 production by AM from HD (p=0.02, Figure 5B). Please note, that LY294002 had to be solved in DMSO, therefore control cells were cultured in the presence of DMSO.

## Discussion

The hallmark of idiopathic pulmonary fibrosis is abundant collagen type I deposition and fibroblast proliferation. On the other hand, in IPF collagen turnover is boosted and collagen cleaving proteases and collagen breakdown products such as collagen type I monomers are increased. While the mature fibrillar collagen type I protein is protected from degradation, monomeric collagen type I is further degraded by matrix metalloproteinases secreted by macrophages and other cells. Several studies [4,13,23] suggested that collagen breakdown products induce distinct immune responses which might promote fibrogenesis. On this background, we analysed immunologic effects on alveolar macrophages induced by collagen monomers. 

AM stimulated with collagen type I monomers produced significantly more CCL18, CCL2, and IL-1ra. This effect was more pronounced in AM from patients with IPF than in those from HD. CCL18 and IL-1ra are considered as markers of M2 macrophage activation and their production is also induced by TH2 cytokines and IL-10 [15]. CCL2 is spontaneously produced by AM and not attributed to M1 or M2 activation. CCL2 production is augmented by IL-10 stimulation and reduced by TH2 cytokine stimulation [24]. We have recently shown that spontaneous CCL18, IL-1ra and CCL2 production of AM is tightly correlated with and increased in patients with fibrotic lung diseases [25]. Thus, AM from patients with IPF showed a hyper-reactivity to stimulation with collagen type I monomers. Further experiments with limited patient numbers revealed that also monomers of collagen type III -V significantly increase CCL18 production by BAL cells and monomers of collagen type III additionally increased IL-1ra production. To our knowledge, we are the first to show that exposure to collagen monomers of human alveolar macrophages exerts a distinct immune effect polarizing macrophages towards alternative activation.

Binding of collagen type I has been described for various cell surface receptors including integrins, discoidin, mannose, and scavenger receptors. Studies by Gowen et al. [26] suggested that under serum-free conditions phagocytosis and adhesion of collagen type I monomers and also other types of collagen monomers by AM is mediated via scavenger receptor type I class A (CD204). Of note, multiple adhesion substrates for scavenger receptors type I class A have been described, including amyloid, glycated and denatured collagens, and proteoglycans present at sites of tissue injury [27]. Furthermore, it has been shown that distinct scavenger receptors are specifically up-regulated by M1 respectively M2 macrophages [15]. CD163 and CD204 were reported as M2 markers, while MARCO is up-regulated in M1 macrophages [15,27-30]. On the background of these findings, we were interested in scavenger receptors expressed by alveolar macrophages in IPF [13]. Our data demonstrate that AM from HD express high levels of CD204, which is further up-regulated in patients with IPF. Of interest, recently an influx of CD204+ macrophages in fibrotic skin of patients with systemic sclerosis was documented [28]. An increase in M2 macrophages expressing CD204 was also noted in tumors [29,30]. In line with the concept that CD204 is a marker of M2 activation [27-30], increased CD204 expression by AM from patients with IPF may reflect M2 pre-activation in IPF. Stimulation with monomeric collagen type I significantly increased CD204 expression of AMs. Thus, the higher CD204 expression of AM in IPF may increase collagen type I induced M2 cytokine production.

The effect of monomeric collagen type I upon M2 marker production by AM was inhibited by neutralizing CD204 antibodies. On the background of our findings, we cannot exclude however, that other receptors may also play a role in collagen type I induced signalling. Noteworthy, scavenger receptor mediated adhesion to other components has also been shown to direct immune responses by altering macrophage activation [31]. Thus, our findings suggest a role of scavenger receptor type I class A (CD204) in directing macrophage activation in pulmonary fibrosis.

There is a vicious circle between fibroblasts and macrophages in IPF amplifying fibrosis [10,13]. On this background, our finding that collagen type I monomers exerts its effects upon macrophages via CD204 and thereby augments M2 activation might have therapeutic impact. However, this effect may not be restricted to collagen type I, since CD204 also recognizes other types of collagens such as type III and IV [26]. In vivo, it was recently shown that alternatively activated macrophages promote pulmonary fibrosis in the bleomycin induced lung fibrosis mouse model [11]. Furthermore, the blockade of CD204 in a mouse model of silica induced pulmonary fibrosis completely abrogated the development of fibrosis [32]. Although there may be other triggers initiating the shift of AM phenotype first, resulting in a kind of hyperresponsiveness to collagen type I monomers, our data suggest one of different mechanisms to uphold the started vicious circle. 

Furthermore, several studies suggest a role of PI3K in alternative macrophage activation [33] triggered by binding of substances to SR-AI resulting in phospho-Akt expression [31,34]. However, the role of PI3K pathway in adhesion to collagen type I has never been addressed. Our data show a high phospho-Akt background expression in AM. Following stimulation with collagen type I, a further significant increase in phospho-Akt expression by AM was observed and this effect was more pronounced in AM from patients with IPF than in HD. Blockade of PI3K activity with LY294002 completely abrogated the immune effects induced by collagen type I monomers. Furthermore, the blockade of PI3K decreased the spontaneous expression of all tested M2 markers. Our data fit well in the concept of a pivotal role of PI3K in alternative macrophage activation [16,32]. Our findings suggest, that the PI3K signalling pathway favours fibrosis mediated not only by its effects on fibroblasts, but also on macrophages. Therapeutic strategies addressing this pathway might be of fundamental interest in fibrotic lung disease.

In conclusion, our data demonstrate that collagen monomers exert a distinct immune effect up-regulating M2 marker and phospho-Akt expression in human AM. Furthermore, we demonstrate a role of CD204 in monomeric collagen type I adhesion by AM. Our data support the recently developed pathogenetic concept of an amplifying loop between macrophages and extracellular matrix [13]. In combination with published in vivo data [9,11,23,32], our studies suggest that CD204 and the PI3K signalling pathway might be interesting targets for new treatment strategies in patients with fibrotic lung disease.
